# 

**Published:** 1874-10

**Authors:** 


### TABLE OF CONTENTS.

ORIGINAL COMMUNICATIONS.

Digitalis—Is it a Cardiac Sedative, or Cardiac Stimulant? By T. Curtis Smith, M.D., - - - - - - - - - - - - - 565

Salicine. By N. D. Tobey, M.D., - - - - - - - - - 579

The Cause of Periodicity in Intermittent Fever. By A. C. Edwards, M.D., - 583

Salicine in Diarrhœa and Dysentery. By J. C. Bishop, M.D. - - - 585

SELECTIONS.

Treatment of Hemorrhagic Malarial Fever. By E. D. McDaniel, A.M., M.D. 588

Puerperal Fever—594. The Value of Pepsin, - - - - - - - 596

REMARKS, GLEANINGS AND EXTRACTS.

More Testimony on the Alcohol Question—597. A New Vaginal Speculum—598. Traumatic Keratitis—600. Treatment of Hay Fever—601. On the Value of High Powers in the Diagnosis of Blood Stains—602. Prolapse of the Vitreous Humor; Iodoform in Venereal Ulcers—603. Doctors and the Poets; Puerperal Amaurosis; Vinegar Bitters—604. Ergot as a Styptic—605. Orchitis; Correction; Incontinence of Urine in Children—606. The Value of Large Injections—607. Reports on Bloodless Operations—608. New Researches on Diabetes; Digestion and Absorption in the Large Intestines—610. The Automatic Man—612. Action of Nitrite of Amyl in Restoring Temporarily a Moribund Patient—613. Influence of Chloroform in Labor upon the Fœtus; Treatment of Chloroform Asphyxiation—615. Atropia in Phthisical Sweating; Treatment of Obstinate Vomiting—616. Iced Cysters in Dysentery; Treatment of Varix by Injections of Chloral Hydrate—617. Bismuth to Prevent Chafing; On the Use of Post-Partum Binders—618. Treatment of Lumbago; Galvanism in Post-Partum Hemorrhage—619. Sedative Power of the Bromide of Potassium; Lead in Subnitrate of Bismuth —620.

EDITORIAL.

Political Honors to Physicians, - - - - - - - - - - - 621

To the Medical Profession of Georgia, - - - - - - - 622

The Physiology of Man—Vol. V., - - - - - - - - - 623

